# Impact of International Atomic Energy Agency support to the development of nuclear cardiology in low-and-middle-income countries: Case of Latin America and the Caribbean

**DOI:** 10.1007/s12350-019-01805-w

**Published:** 2019-07-08

**Authors:** C. Gutierrez-Villamil, A. Peix, P. Orellana, I. Berrocal, R. Ramirez, E. Estrada-Lobato, D. Paez

**Affiliations:** 1 Servicio de Medicina Nuclear, Fundación Cardioinfantil – Instituto de Cardiología, Bogotá, Colombia; 2 Escuela de Medicina y Ciencias de la Salud, Universidad del Rosario, Bogotá, Colombia; 3 Nuclear Medicine Department, Institute of Cardiology, 17 No. 702, Vedado, CP 10 400 Havana, Cuba; 4 Nuclear Medicine Unit, Radiology Department, Pontificia Universidad Catolica de Chile, Santiago, Chile; 5 Hospital San Juan de Dios, San Jose, Costa Rica; 6 Technical Cooperation Section for Latin America and the Caribbean, International Atomic Energy Agency, Vienna, Austria; 7 Nuclear Medicine and Diagnostic Imaging Section, International Atomic Energy Agency, Vienna, Austria

**Keywords:** Nuclear cardiology, cardiac imaging, cardiovascular disease, Latin America, Caribbean

## Abstract

Cardiovascular diseases (CVDs) are the leading cause of death in Latin America and the Caribbean (LAC) region as well as worldwide. Lifestyle, nutritional habits and the upsurge of obesity have contributed to the increase in the prevalence of CVDs in the region. The role of nuclear cardiology in the management of patients with CVDs is well established. Particularly, myocardial perfusion imaging is widely used in LAC countries and has been increasingly integrated into the healthcare systems in the region for the diagnosis of coronary artery disease, risk stratification and to guide patient management. In its role to support countries around the world to address their health needs through the peaceful applications of nuclear techniques, the International Atomic Energy Agency (IAEA) has provided assistance to the LAC region for the establishment and strengthening of the nuclear cardiology practice. To that extent, the IAEA provides support in building capacities of multidisciplinary teams of professionals, the provision of medical equipment and the promotion of communication and exchange of knowledge among the different stakeholders. In addition, the IAEA encourages the participation of nuclear medicine centers in international multi-center research studies. In this paper, we present some of the projects through which the IAEA has supported the LAC region, including regional technical cooperation projects and coordinated research projects related to cardiology within the current multimodality approach to cardiac imaging.

**Electronic supplementary material:**

The online version of this article (10.1007/s12350-019-01805-w) contains supplementary material, which is available to authorized users.

## Introduction

The Latin American and Caribbean (LAC) region has a population of over 600 million inhabitants, with almost 80% concentrated in urban areas. Literacy is above 91%, life expectancy reaches a mean of 75 years, and the population is growing at an average of 1.1% annually.^1^ Public expenditure in healthcare as a percentage of gross national product varies between 4.4 and 10.9.^2^

In the last decades, the region has experienced several social, demographic, and economic changes that have had a significant impact on public health. Aging, globalization, urbanization and the rise in obesity and physical inactivity in the region have made cardiovascular diseases the leading cause of death and disability.^3^ Cardiovascular diseases (38%), cancer (25%), respiratory diseases (9%), and diabetes (6%) are the four leading causes of death due to non-communicable diseases (NCDs)^4^ which are responsible for approximately 75% of deaths.^5^

Cardiovascular diseases account for most NCDs deaths, with 17.9 million deaths worldwide annually, followed by cancers with 9.0 million, according to the World Health Organization (WHO).^6^ Cardiovascular diseases constitute the main cause of death in the region, especially ischemic heart disease, given the high prevalence of cardiovascular risk factors (e.g. diabetes mellitus, obesity, and smoking), and there is concern over a growing epidemic and potential for higher mortality related to cardiovascular diseases. In this context, diagnostic nuclear medicine (NM) techniques can play a significant role and be of great impact in the cost-effective management of most of these patients.

According to the WHO, NCDs are fundamentally a development and socioeconomic issue, striking both rich and poor people, but inflicting more ill health and other consequences on the poor in all countries. The LAC region has been recognized as the one with the highest socioeconomic disparities, a status that can contribute to the high mortality due to NCDs, including cardiovascular diseases, diabetes, and cancer.

The practice of NM in the LAC region has experienced important growth in the last decade. However, there is great heterogeneity among countries regarding the availability of technology and human resources. According to data published in 2015^7^, the total number of gamma cameras in the region is 1,231, with an average of 2.16 per million inhabitants.

Over 90% of the cameras are SPECT, 7.6% of which have hybrid technology. There were 161 operating PET or PET/CT in 12 Member States (MS), representing a rate of 0.3 per million people. Most NM centres belong to the private health system and are in capitals or major cities. Nearly 94% of the procedures are diagnostic; PET studies represent about 4% of the total.

Only four countries have the capability of assembling ^99^Mo–^99m^Tc generators, and two countries produce ^99m^Tc from nuclear reactors. Cold kits are produced in some countries and therapeutic agents are mostly imported from outside the region. There were 35 operative cyclotrons.

In relation to human resources there was one physician per gamma camera, 1.6 technologists per gamma camera, 0.1 medical physicists per centre, and approximately 0.1 radiopharmacist per centre.

Nuclear cardiology utilization in the region is very heterogeneous and still, in general terms, underutilized. But the future of NM in the LAC region is promising, with great potential and possibilities. Some of the most important factors driving the region toward greater homogeneity in the availability and application of NM and bridging the gaps between countries are continuous training and clinician awareness of the importance of NM in managing prevalent diseases in the region, as well as the increased building of capacity, public–private partnerships, government commitment and the continuous and strong support from international organizations such as the International Atomic Energy Agency (IAEA) through national and regional projects.

The main objective of the IAEA programme in Human Health is to enhance the capabilities in MS to address needs related to the prevention, diagnosis and treatment of health problems through the application of nuclear techniques. The IAEA has provided continuous support to 21 countries of the region, improving capacity building and technology according to individual country needs.

Many tools are available for IAEA support. In this article, we present the benefits obtained through three Regional Latin American (RLA) projects and nine coordinated research projects (CRPs) related to cardiology within the present multimodality approach in cardiac imaging.

## Regional Projects

Three regional projects addressing cardiac diseases have been successfully implemented in the region between 2009 and 2018:

- RLA 6063: Improving the attention of patients with cardiac diseases and cancer patients by the strengthening of nuclear medicine techniques in Latin America and the Caribbean region (2009 to 2013).
- RLA 6070: Harmonization of nuclear cardiology techniques in patients with congestive heart failure, with emphasis on chagasic cardiomyopathy (2012 to 2013).
- RLA 6078: Facing the high incidence of cardiovascular diseases in Latin America and the Caribbean through nuclear cardiology (2016 to 2018).

Table 1 shows the main outputs obtained through these projects, which addressed the capacity building through fellowships, regional training courses and expert missions. In addition, several papers were published regarding the value of imaging techniques with emphasis on nuclear cardiology, their standardization in the region and the specificities of the cardiovascular health situation in the region.^7^–^11^

**Table 1:** Outputs obtained in the LAC region through regional projects

Project	Regional training courses	Fellowships	Expert missions	Publications	Procurements
RLA 6063	2	–	14	1	–
RLA 6070	4	5	15	1	7*
RLA 6078	5	2	17	3	5*

*LAC* Latin America and the Caribbean, *RLA* Regional Latin America

*Emory cardiac toolbox licenses

Table 2 presents the number of professionals trained and missions of experts received per country participating in the different projects.

**Table 2:** Number of professionals trained and experts sent by IAEA per country participating in the projects

	Country	Professionals trained	Expert missions
1	ARGENTINA**	16	1
2	BOLIVIA***	19	3
3	BRAZIL***	24	12
4	CHILE***	32	4
5	COLOMBIA***	86	4
6	COSTA RICA**	11	9
7	CUBA***	157	3
8	DOMINICAN REPUBLIC **	16	0
9	ECUADOR**	46	1
10	EL SALVADOR*	2	0
11	HAITI*	0	0
12	JAMAICA*	0	0
13	MEXICO**	49	0
14	NICARAGUA**	6	3
15	PANAMA*	8	0
16	PARAGUAY**	8	1
17	PERU***	24	2
18	URUGUAY**	13	0
19	VENEZUELA**	20	2
TOTAL		537	46

*IAEA* International Atomic Energy Agency

*Participation in one project

**Participation in two projects

***Participation in three projects

## Coordinated Research Projects

The purpose of the IAEA’s CRPs is to promote the acquisition and dissemination of knowledge and technologies generated from the peaceful uses of atomic energy; encourage and facilitate research in MS, in particular low- and middle-income countries (LMICs); stimulate the growth of nuclear sciences and technologies in LMICs, and promote the research activities within the MS and create a framework for supporting scientific and technical exchanges between countries (LMICs and high-income countries). The CRPs allow researchers from around the world to work with an integrated approach on basic and clinical scientific topics of interest for the international community, offering a real-world approach to the problem and helping to disseminate protocols and algorithms shown to be useful in clinical practice.

Since 2000, the IAEA has implemented nine CRPs in the field of cardiology, all with the participation of LAC centers:

- Intravascular radionuclide therapy using liquid beta-emitting radiopharmaceuticals to prevent restenosis. Chile, Colombia, Cuba and Uruguay were part of the project.
- Nitrate Augmented Myocardial Imaging for Assessment of Myocardial Viability. With the participation of centres from Cuba and Uruguay.
- Role of nuclear cardiology in ischemia assessment with exercise imaging in asymptomatic diabetics (IAEA Diabetes). Centers from Argentina, Chile, Colombia, Cuba and Uruguay were involved in the research.^12^–^16^
- Performance of rest MPI in the management of acute chest pain in the emergency room. (Four LAC centres were involved including two from Brazil, and one from Chile and Cuba).^17^,^18^
- Assessment of left ventricular function in coronary artery disease with nuclear techniques. With the participation of Brazil, Cuba, Uruguay and Venezuela.^19^,^20^
- Functional compared to anatomical imaging in the initial evaluation of patients with suspected coronary artery disease: an international, multicentre, randomized controlled trial (IAEA SPECT/CTA study). With centers from Brazil and Mexico.^21^,^22^
- Value of intraventricular synchronism assessment by gated-SPECT myocardial perfusion imaging in the management of heart failure patients submitted to cardiac resynchronization therapy (VISION-CRT) (centers from Brazil, Chile, Colombia, Cuba and Mexico participated in the study).^23^–^26^
- Value of Gated-SPECT MPI for Ischemia-Guided PCI of non-culprit vessels in STEMI Patients with Multivessel Disease after primary PCI (on-going project that includes researchers from Brazil, Cuba and Mexico).
- Imaging in Cardio-oncology Study (iCOS) (on-going project that includes three centres from two countries: Brazil and Cuba.^2^

## How the Lessons Learned Can Contribute to Good Quality Nuclear Cardiology in the Region

To maximize the results and obtain the highest benefits, the resources from different regional projects have been merged. The coordinated work and integration of the regional projects have proven to be very useful for the LAC region, thus allowing a greater impact in capacity building, strengthening the nuclear cardiology and improving quality of care for the benefit of the patients. This integration was made possible due to the cooperation among participant countries and the coordination of the projects done by the IAEA technical officers, who have an in-depth knowledge of the projects allowing the integration of activities among them.

It is important to point out that the results obtained from the CRPs and regional projects have been conveniently disseminated through scientific publications, with an increase in the number of papers published in peer-reviewed medical journals, and therefore, with more visibility for the scientific work sponsored by the IAEA in the region. It is also a means to promote continuing education for the professional community and improve the quality of the clinical practice.

In addition, two other important achievements are: the commitment of the participants in the different activities to disseminate the acquired knowledge, which is of pivotal importance to improve the competencies of the professionals in the region; raising awareness of the clinical applications of nuclear cardiology, achieved through the participation of international IAEA experts in national and regional meetings. In this sense, the collaboration of IAEA experts and those from medical associations such as the American Society of Nuclear Cardiology (ASNC), Asociación Latinoamericana de Sociedades de Biología y Medicina Nuclear (ALASBIMN) and the European Association of Nuclear Medicine (EANM) has been essential.

Several interinstitutional, national and international agreements have improved the cooperation among countries, as evidenced by the activities of scientific professional organizations that play an important role in educational and strategic support.

A key aspect has been the use of a common language among most countries in the region, which facilitates scientific exchange, information access, and continuing professional education.

On the other hand, the use of new information and communication technologies for education and training, such as online trainings, webinars and interactive e-learning materials, is of paramount importance to achieve dissemination of the information in the region with a broader outreach. In every case, the participation of the referral practitioners (cardiologists, internal medicine physicians, emergency doctors, and cardiac surgeons, among others) in the different educational activities, in addition to the nuclear medicine specialists and nuclear cardiologists, has been considered an important achievement, since it allows better knowledge of the clinical applications and appropriate use of the nuclear cardiology techniques.

Finally, the concept of multimodality imaging has been included in the different educational activities and publications related to these projects, and this significantly contributes to improving the diagnostic and prognostic value of the imaging techniques in cardiac patients in a cost-effective way, considering the appropriate and justified use of each medical imaging modality in the different clinical scenarios, the adequate radiological protection and the quality management.

## Hurdles and Possibilities of Solution Through Regional Cooperation

The development of nuclear cardiology studies and other advanced imaging modalities in Cardiology are hampered in the region by several factors such as high cost of equipment, lack of sustainable equipment maintenance and service, insufficient cardiac-specific training and, in some cases, lack of awareness of cardiologists or other referring physicians of the clinical applications of nuclear cardiology.^9^,^27^ It is necessary to include training in hybrid imaging (SPECT-CT and PET-CT), as well as in cardiac CT. Professionals involved in nuclear cardiology should increase their participation in training programs, certification courses, fellowships, and webinars, where the support of IAEA, ALASBIMN and ASNC, as well as national professional medical organizations, is of crucial importance.

Another hurdle to overcome is related to the supply of radioisotopes/radiopharmaceuticals, both for SPECT and PET studies. With this aim, programs that could make isotopes, drugs, and equipment available at a lower cost for LMICs have been proposed.^9^,^27^ Continued cooperation among countries of the region would also be very helpful. It is important to point out that PET applications in cardiology should become available in the region, not only for viability assessment purposes with ^18^F-FDG, but also for myocardial blood flow evaluation for the detection of multivessel disease and microvascular angina, as well as for the assessment of patients with infective endocarditis and infection of cardiac devices.

Cardiovascular disease is the main cause of women’s death both in LAC and worldwide,^11^ and coronary risk factors and ischemic heart disease (IHD) continue increasing in the region. Thus, to include more women in research trials as well as to implement new regional projects and national and regional health policies aiming to raise awareness about IHD in women and how to face the problem, is of paramount importance. Emphasis should be made both on prevention and proper medical and interventional management. In this context, it is important to reinforce the value of imaging techniques, including nuclear cardiology tests.

## Role of Multimodality in Cardiology

At present, multimodality imaging in clinical practice implies a shift from a comparison of technology to an integration of diagnostic strategies, offering the most clinically effective and cost-effective testing for an optimal patient outcome. Thus, it is imperative to carefully evaluate and select the most appropriate study (e.g. echo, SPECT, PET, cardiac computed tomography angiography, and cardiac magnetic resonance imaging), to be performed in each individual patient to optimize diagnosis, risk stratification, and ischemia-guided therapies in IHD patients.

A very important part of this approach includes education and cross-training of the imaging specialists, as well as the conception of a relatively new figure: the imaging consultant. In this sense, the contribution of IAEA, ASNC, ALASBIMN and other international, regional and national professional medical organizations and experts is extremely important.

## Opportunities for Improvement of Nuclear Cardiology Practices and Radiation Exposure in Latin America and the Caribbean

In 2013 the IAEA conducted the *International Atomic Energy Agency Nuclear Cardiology Protocols Study* (INCAPS), to characterize patient radiation doses from MPI, and to determine the use of radiation-optimizing “best practices” (BPs). These BPs were identified a priori and a radiation-related Quality Index (QI) was defined. The QI of an institution was determined according to the number of BPs to which they adhered to and complied with.^28^,^29^ Among the 308 centers worldwide, 36 centers from 10 LAC countries joined the INCAPS study. It was noted that (1) the LAC centers have the highest median radiation dose (12.1 mSv), (2) a wide variation in laboratory mean effective dose (ED) with only 11% of the institutions achieving a median ED ≤ 9 mSv, (3) and the lowest proportion of laboratories with QI score of at least 6.

These findings have provided a better understanding of the current practices of nuclear cardiology in LAC countries, as well as the opportunity to identify areas in which the IAEA can support to improve safety and quality of care, designing projects tailored to the specific needs of the region.

## Conclusion

The LAC region is experiencing a great burden of cardiovascular diseases, responsible for the highest mortality in the region, both in women and men. A well-oriented prevention and proper utilization of cardiac imaging, including nuclear cardiology, within a multimodality approach, are essential to address this health challenge. National, regional and international cooperation, including support from scientific professional organizations such as ASNC, EANM and ALASBIMN and United Nations organizations such as the IAEA or WHO, as well as government commitment, are strategically important to face the increasing burden of CVDs and to build capacities in the management of patients and to further strengthen the practice of nuclear cardiology.

## New Knowledge Gained

A well-oriented prevention and proper utilization of cardiac imaging, including nuclear cardiology, within a multimodality approach, are essential to address the great burden of cardiovascular diseases, responsible for the highest mortality in the region, both in women and men. National, regional and international cooperation, including support from scientific professional organizations and United Nations organizations such as the IAEA and WHO, as well as government commitment, are key factors.

## Electronic supplementary material

Below is the link to the electronic supplementary material.

Supplementary material 1 (PPTX 166 kb)

## Abbreviations

CAD: Coronary artery disease
CRP: Coordinated research project
CVDs: Cardiovascular diseases
IAEA: International Atomic Energy Agency
LAC: Latin American and Caribbean
LMIC: Low- and middle-income country
MPI: Myocardial perfusion imaging
NC: Nuclear cardiology
NCDs: Non-communicable diseases
RLA: Regional Latin American
