# Supplementary material for: Impact of International Atomic Energy Agency support to the development of nuclear cardiology in low-and-middle-income countries: Case of Latin America and the Caribbean
Source: J Nucl Cardiol. 2019 Jul 8;26(6):2048–54. doi: 10.1007/s12350-019-01805-w (PMC6908563; doi:10.1007/s12350-019-01805-w)
Supplement: Supplementary file 1 — Supplementary material 1 (PPTX 166 kb) [file 12350_2019_1805_MOESM1_ESM.pptx]

## Slide 1
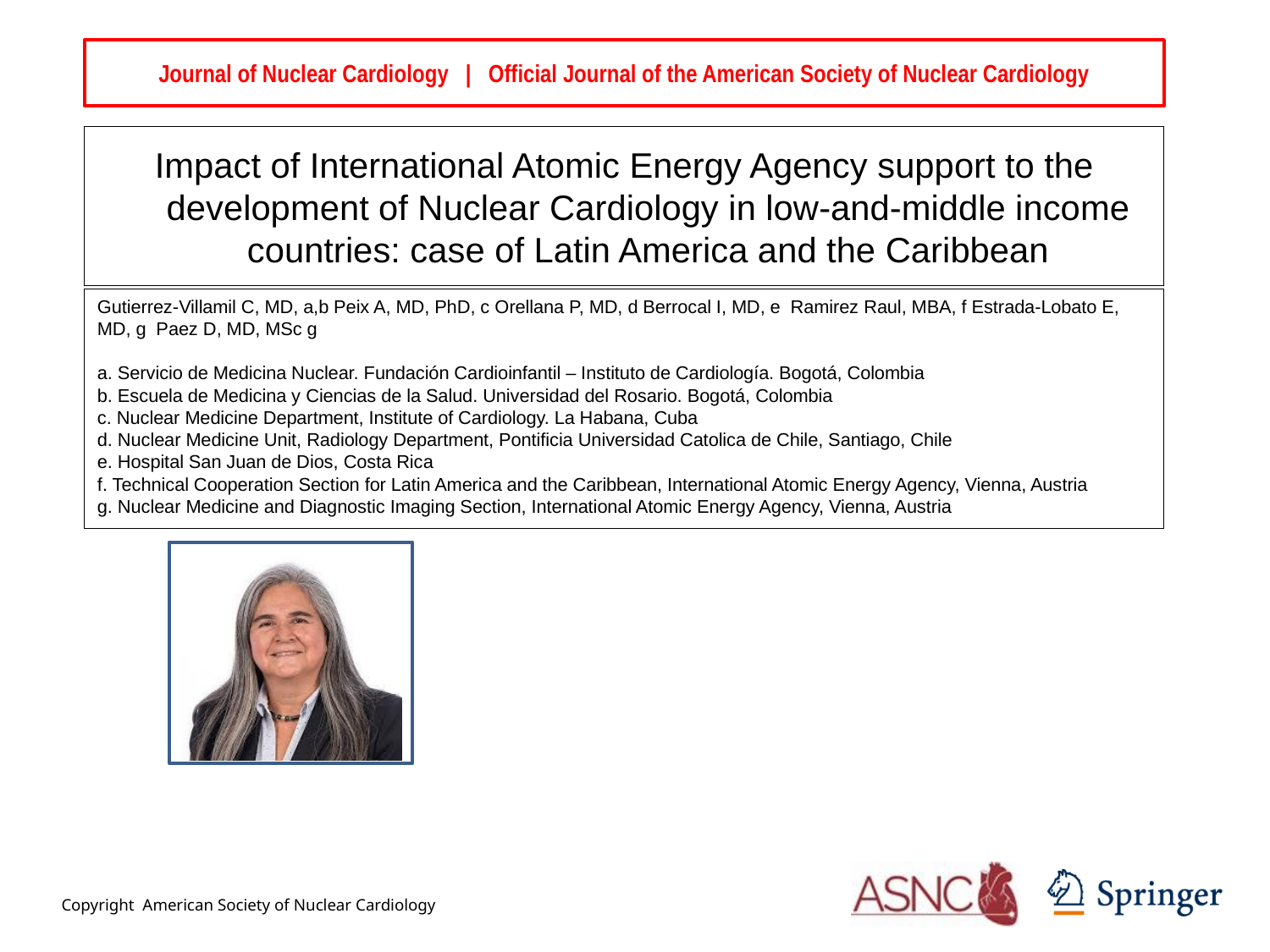

Journal of Nuclear Cardiology | Official Journal of the American Society of Nuclear Cardiology
# Impact of International Atomic Energy Agency support to the development of Nuclear Cardiology in low-and-middle income countries: case of Latin America and the Caribbean
Gutierrez-Villamil C, MD, a,b Peix A, MD, PhD, c Orellana P, MD, d Berrocal I, MD, e Ramirez Raul, MBA, f Estrada-Lobato E, MD, g Paez D, MD, MSc g
a. Servicio de Medicina Nuclear. Fundación Cardioinfantil – Instituto de Cardiología. Bogotá, Colombia
b. Escuela de Medicina y Ciencias de la Salud. Universidad del Rosario. Bogotá, Colombia
c. Nuclear Medicine Department, Institute of Cardiology. La Habana, Cuba
d. Nuclear Medicine Unit, Radiology Department, Pontificia Universidad Catolica de Chile, Santiago, Chile
e. Hospital San Juan de Dios, Costa Rica
f. Technical Cooperation Section for Latin America and the Caribbean, International Atomic Energy Agency, Vienna, Austria
g. Nuclear Medicine and Diagnostic Imaging Section, International Atomic Energy Agency, Vienna, Austria
Head shot of author
required
Copyright American Society of Nuclear Cardiology

## Slide 2
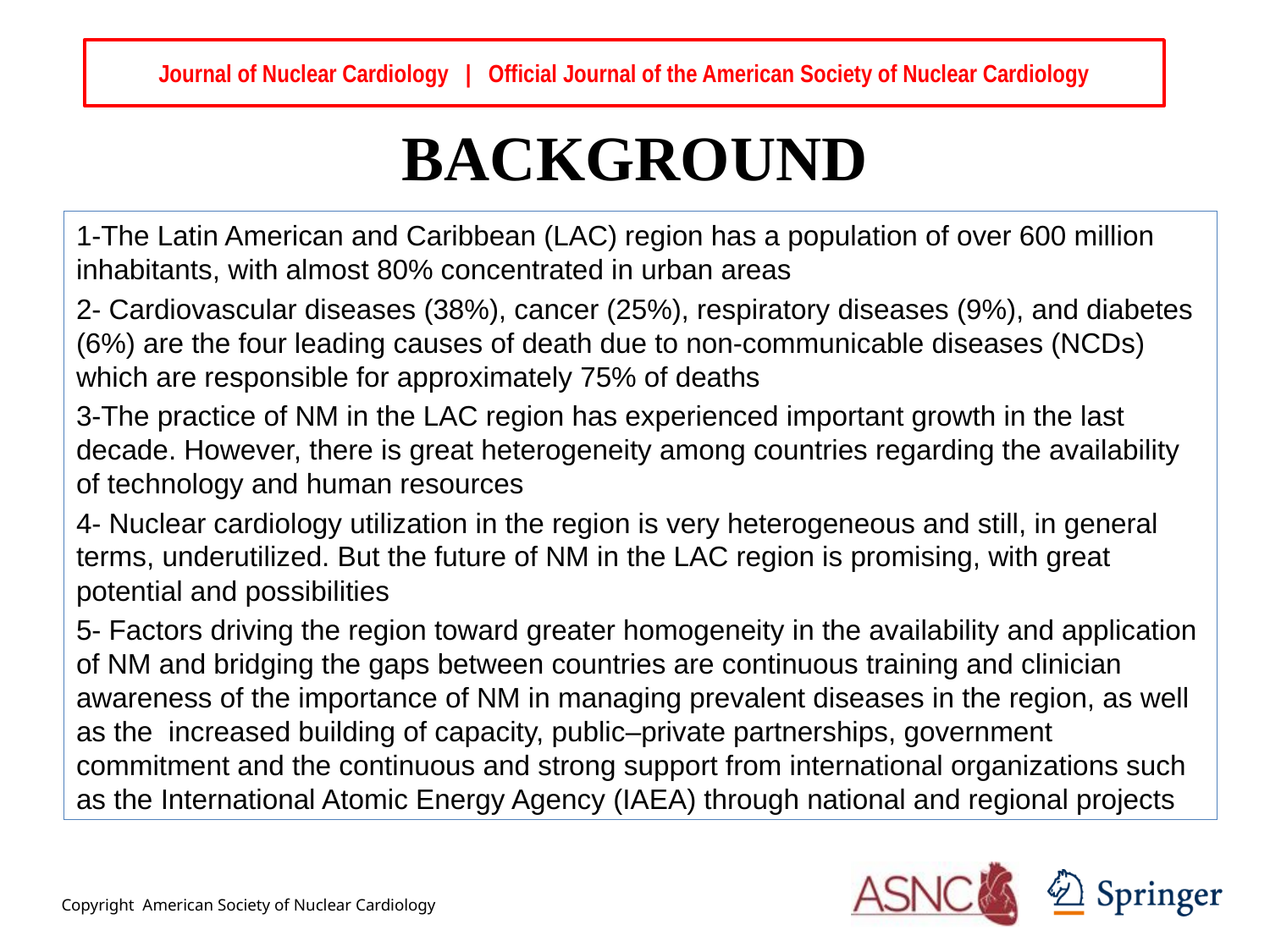

Journal of Nuclear Cardiology | Official Journal of the American Society of Nuclear Cardiology
# BACKGROUND
1-The Latin American and Caribbean (LAC) region has a population of over 600 million inhabitants, with almost 80% concentrated in urban areas
2- Cardiovascular diseases (38%), cancer (25%), respiratory diseases (9%), and diabetes (6%) are the four leading causes of death due to non-communicable diseases (NCDs) which are responsible for approximately 75% of deaths
3-The practice of NM in the LAC region has experienced important growth in the last decade. However, there is great heterogeneity among countries regarding the availability of technology and human resources
4- Nuclear cardiology utilization in the region is very heterogeneous and still, in general terms, underutilized. But the future of NM in the LAC region is promising, with great potential and possibilities
5- Factors driving the region toward greater homogeneity in the availability and application of NM and bridging the gaps between countries are continuous training and clinician awareness of the importance of NM in managing prevalent diseases in the region, as well as the increased building of capacity, public–private partnerships, government commitment and the continuous and strong support from international organizations such as the International Atomic Energy Agency (IAEA) through national and regional projects
Copyright American Society of Nuclear Cardiology

## Slide 3
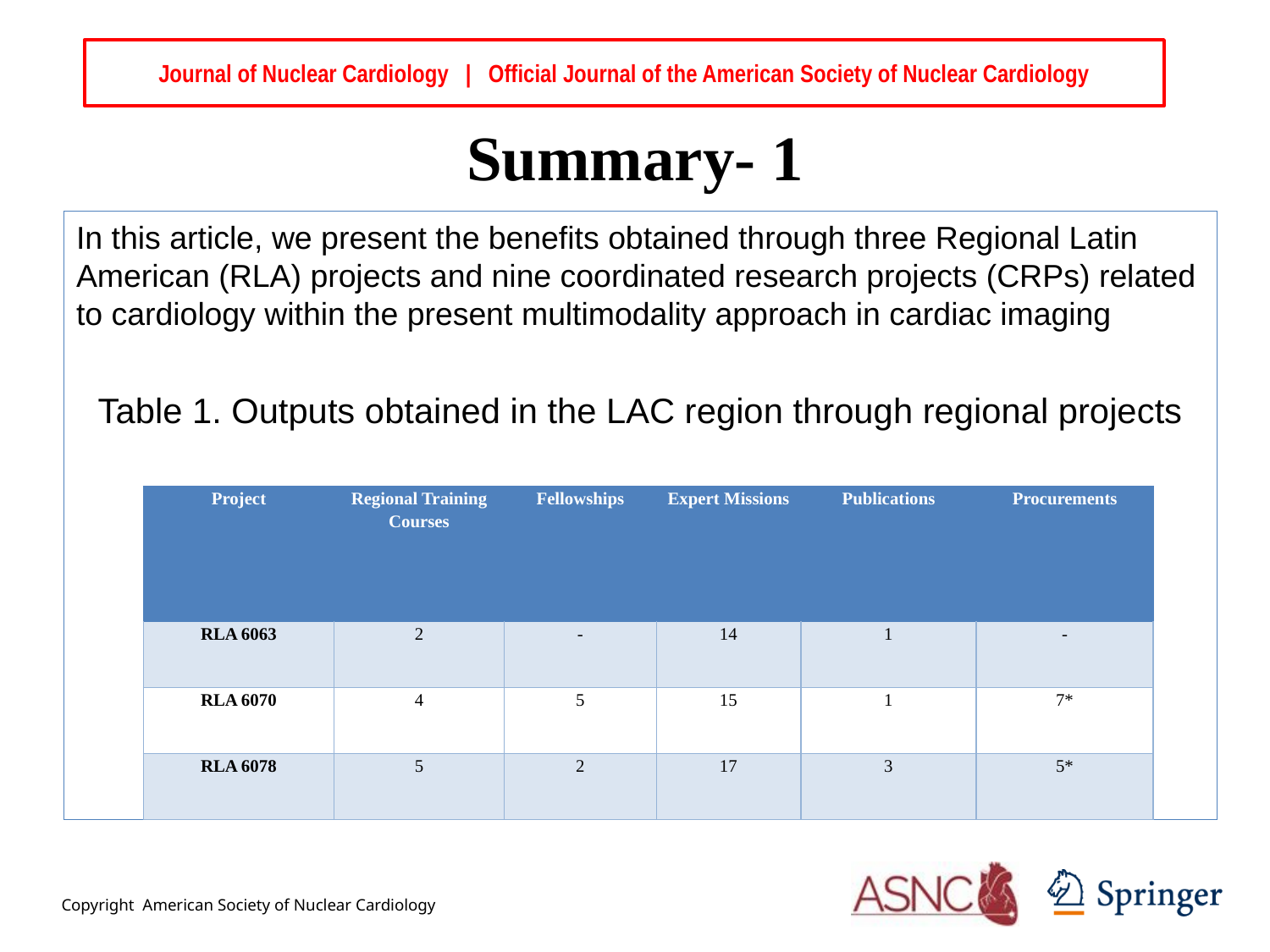

Journal of Nuclear Cardiology | Official Journal of the American Society of Nuclear Cardiology
# Summary- 1
In this article, we present the benefits obtained through three Regional Latin American (RLA) projects and nine coordinated research projects (CRPs) related to cardiology within the present multimodality approach in cardiac imaging
Table 1. Outputs obtained in the LAC region through regional projects
| Project | Regional Training Courses | Fellowships | Expert Missions | Publications | Procurements |
| --- | --- | --- | --- | --- | --- |
| RLA 6063 | 2 | - | 14 | 1 | - |
| RLA 6070 | 4 | 5 | 15 | 1 | 7\* |
| RLA 6078 | 5 | 2 | 17 | 3 | 5\* |
Copyright American Society of Nuclear Cardiology

## Slide 4
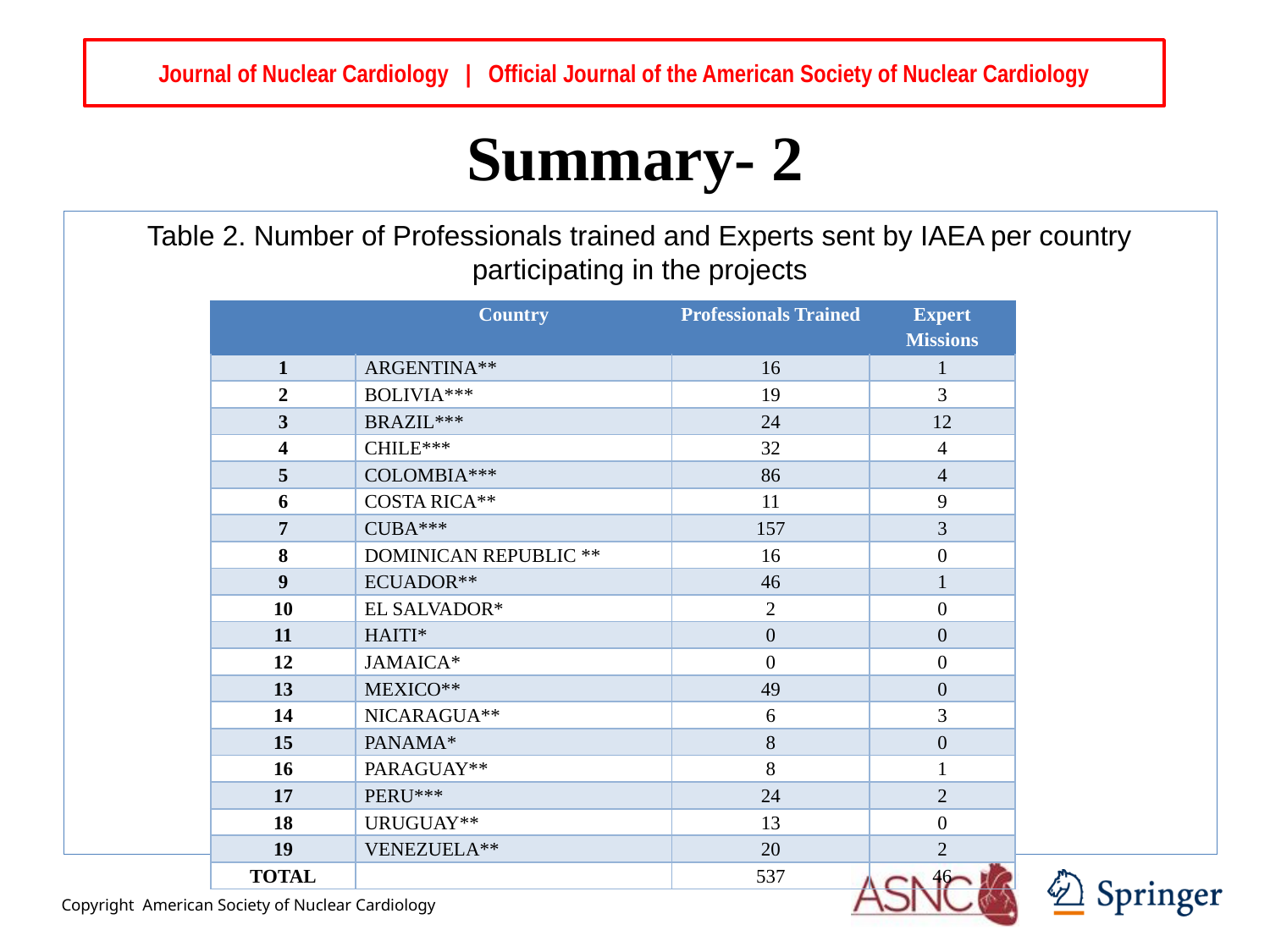

Journal of Nuclear Cardiology | Official Journal of the American Society of Nuclear Cardiology
# Summary- 2
Table 2. Number of Professionals trained and Experts sent by IAEA per country participating in the projects
| | Country | Professionals Trained | Expert Missions |
| --- | --- | --- | --- |
| 1 | ARGENTINA\*\* | 16 | 1 |
| 2 | BOLIVIA\*\*\* | 19 | 3 |
| 3 | BRAZIL\*\*\* | 24 | 12 |
| 4 | CHILE\*\*\* | 32 | 4 |
| 5 | COLOMBIA\*\*\* | 86 | 4 |
| 6 | COSTA RICA\*\* | 11 | 9 |
| 7 | CUBA\*\*\* | 157 | 3 |
| 8 | DOMINICAN REPUBLIC \*\* | 16 | 0 |
| 9 | ECUADOR\*\* | 46 | 1 |
| 10 | EL SALVADOR\* | 2 | 0 |
| 11 | HAITI\* | 0 | 0 |
| 12 | JAMAICA\* | 0 | 0 |
| 13 | MEXICO\*\* | 49 | 0 |
| 14 | NICARAGUA\*\* | 6 | 3 |
| 15 | PANAMA\* | 8 | 0 |
| 16 | PARAGUAY\*\* | 8 | 1 |
| 17 | PERU\*\*\* | 24 | 2 |
| 18 | URUGUAY\*\* | 13 | 0 |
| 19 | VENEZUELA\*\* | 20 | 2 |
| TOTAL | | 537 | 46 |
Copyright American Society of Nuclear Cardiology

## Slide 5
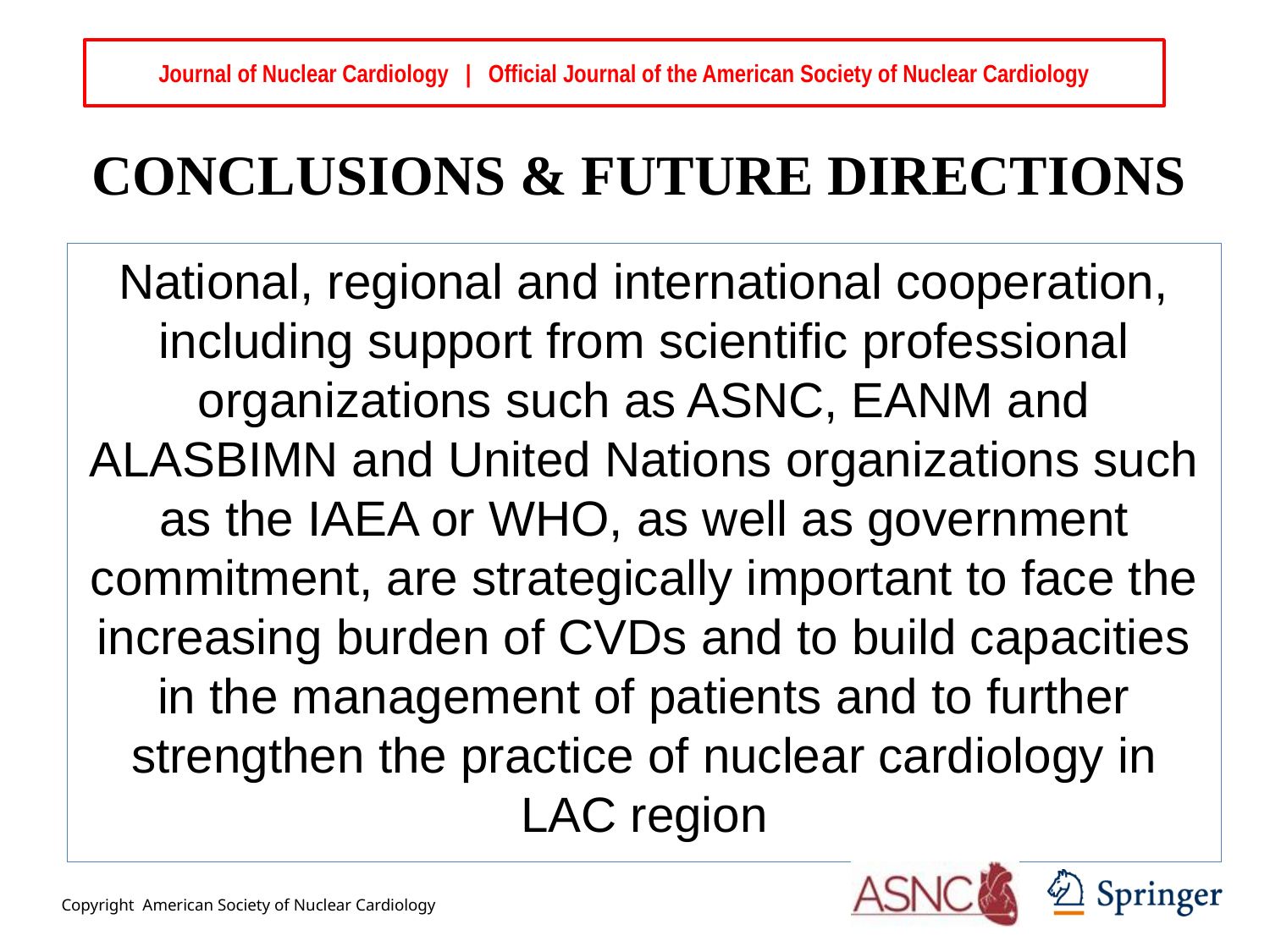

Journal of Nuclear Cardiology | Official Journal of the American Society of Nuclear Cardiology
# CONCLUSIONS & FUTURE DIRECTIONS
National, regional and international cooperation, including support from scientific professional organizations such as ASNC, EANM and ALASBIMN and United Nations organizations such as the IAEA or WHO, as well as government commitment, are strategically important to face the increasing burden of CVDs and to build capacities in the management of patients and to further strengthen the practice of nuclear cardiology in LAC region
Copyright American Society of Nuclear Cardiology
